# Faculty perspectives on supervising endocrinology residents in the longitudinal clinic setting

**DOI:** 10.1016/j.jcte.2025.100385

**Published:** 2025-02-28

**Authors:** Isabella Albanese, Katherine Drummond, Vanessa Tardio, Diana Dolmans

**Affiliations:** a McGill University Health Centre, Division of Endocrinology and Metabolism, Canada; b PGY2 Resident, McGill University, Department of Internal Medicine, Canada; c Educational Sciences, Chair Department of Educational Development and Research, Maastricht University, Department of Educational Development and Research, School of Health Professions Education, the Netherlands

**Keywords:** Endocrinology, Education, Residents, Longitudinal clinic, Supervision

## Abstract

**Introduction:**

Longitudinal clinic supervision is complex but key to training residents, such as endocrinology residents. Supervisors report difficulties providing learners with the right balance of supervision and autonomy. Cognitive apprenticeship theory applies to teaching in workplace-based settings, such as the endocrinology longitudinal clinic. While this theory identifies effective strategies for supervising medical students in longitudinal settings, little is known about how and why supervisors adapt their strategies when offering longitudinal supervision to endocrinology residents.

**Objective:**

To understand supervisors’ perspectives on supervision of endocrinology residents over time in the longitudinal clinic.

**Methods:**

This is a qualitative study set in the endocrinology division of a Canadian multi-site academic centre in 2023–2024 using semi-structured interviews of endocrinology longitudinal clinic supervisors. Transcripts were analyzed using inductive and deductive thematic analysis, with cognitive apprenticeship theory as a sensitizing concept.

**Results:**

Five main themes were identified articulating how participants perceive their supervision and its changes over time. These include supervisors treating residents as colleagues and making clinic experiences reflect real life when possible. Supervisors also tailor clinic experiences to resident needs and career goals. They provide residents with support but also autonomy to execute their own management plans even if discordant with that of supervisors provided that patient safety is maintained. Similarly, supervisors encourage residents to take patient ownership. Together, these strategies enable supervisors to support resident development into independent professional endocrinologists.

**Conclusion:**

Overall, supervisors try to encourage residents to self-regulate their learning, but also offer support and autonomy to enhance their longitudinal growth and development.

## Highlights

• Little is known about how and why supervisors adapt their supervision of endocrinology residents in longitudinal clinic.
• This study interviews supervisors on their perspectives on supervising endocrinology residents in longitudinal clinic.
• Supervisors treat residents as colleagues and make clinic experiences reflect real life when possible.
• Residents are given support but also autonomy to execute their own plans even if discordant with that of supervisors.
• Overall, supervisors try to encourage residents to self-regulate their learning, but also offer support and autonomy.

## Introduction

Longitudinal clinic is an accreditation requirement for endocrinology residency programs in the United States and Canada [1], [2]. Despite this, studies informing supervision of endocrinology residents and subspecialty residents in longitudinal clinics overall are lacking. Supervisors report difficulties in providing learners with the right balance of supervision and support [3], [4]. This creates tensions when learners perceive learning opportunities to have an imbalance of autonomy and support [5]. Learner perceptions of what makes supervision effective vary by level of training [6], [7]. While students early in training report valuing spoon-feeding, final year students and junior residents value feedback and role-modeling [7]. Furthermore, third-year residents and above reported needing more active, self-directed learning [7]. Research is needed to explore supervisor perceptions of supervising subspecialty residents such as endocrinology residents to further understanding of how to meet their learning needs.

Theoretical Framework

Situated learning understands learning as social, contextual and embedded in meaningful activities [8]. It is integral to continuity in medical education as it underpins the social relationships inherent to learning with longitudinal supervision [9], [10]. Cognitive apprenticeship theory is the instructional model for situated learning and applies to supervision of authentic activities with small teacher-to-learner ratios [11], [12]. The cognitive apprenticeship theory principles relevant to this study are method and sequencing [11]. Method refers to how teachers promote learning and comprises of six strategies: modeling, coaching, scaffolding, articulation, reflection and exploration. Sequencing is the order of learning activities over time considering task complexity and diversity [11]. This study uses the method and sequencing principles to determine how cognitive apprenticeship theory applies to longitudinal clinic supervision in endocrinology residency.

Cognitive apprenticeship theory is a validated concept for evaluating clinical teaching of students and has inspired faculty development [13], [14]. It informs a theoretical model of clinical supervision in which teachers use strategies of modeling and scaffolding early on, followed by coaching, followed by articulation and exploration to stimulate student self-directed learning [15]. This model fit the experiences of teachers in longer clinical rotations involving more experienced students [15]. Thus, cognitive apprenticeship theory is relevant to study longitudinal supervision of subspecialty residents.

The study objective is to understand supervisor perspectives on supervision of endocrinology residents over time in longitudinal clinic through the lens of cognitive apprenticeship theory.

## Methods

Setting

The study setting is the endocrinology division of a Canadian multi-site academic center in 2023–2024. Endocrinology residency is a two-year program completed after a three-year internal medicine residency. Longitudinal clinic is a weekly half-day endocrinology clinic over the last eighteen months of residency, done under one supervisor for eighteen months or two supervisors, each for nine consecutive months.

Sampling

Participants were identified using purposive sampling among current and recent endocrinology longitudinal clinic supervisors and recruited via e-mail. There were fourteen identified longitudinal clinic supervisors in the last five years at this centre, twelve of whom were interviewed. Participation was voluntary. All participants provided written informed consent.

Protocol

This is a qualitative study using semi-structured interviews of participants. Interviews were conducted in-person or online. Interview guide key questions are based on the cognitive apprenticeship theory method principle strategies of modeling, scaffolding, coaching, articulation, exploration and reflection [11]. Each question asks about changes over time to reflect the cognitive apprenticeship theory sequencing principle [11]. The interview guide was pilot-tested and adjusted (Appendix 1: interview guide). Interviews were audio-recorded, transcribed and anonymized.

Analysis

Thematic analysis was performed using a combination of deductive analysis using cognitive apprenticeship theory as a sensitizing concept and inductive analysis in keeping with interpretive description [11], [16]. Transcripts were analyzed using an iterative constant comparative approach. After every three interviews, the interview guide was adjusted to further explore identified themes from data analysis. Two authors reviewed transcripts independently and coded using ATLAS.ti software version 23.4.0, while checking interpretation for consistency. The authors kept memos during analysis to review trends and detect patterns and themes. Themes and quotes were discussed amongst all authors. Interviews were conducted until sufficiency, such that no new themes were discovered.

Reflexivity

All authors are clinicians or health professions researchers interested in supporting learners in the workplace. We acknowledge our subjectivity, discussed findings and critically reflected on them from these different perspectives to address our ongoing biases in data collection, analysis and interpretation.

## Results

Twelve interviews were conducted among supervisors with an average of fifteen years of supervision experience, ranging from three to thirty-four years. Five themes were identified articulating how participants perceive their supervision and how it changes over time: “treat the resident as a colleague and create a partnership”, “make clinic reflect real life by increasing volume and administrative tasks “tailor clinic to resident needs and career goals”, “let residents make decisions and learn from them” and “let residents take patient ownership”.

Theme 1: Treat the resident as a colleague and create a partnership

Supervisors perceive endocrinology residents to be equal team members. They view them as future colleagues and describe valuing their input and how the clinic allows them to learn from each other.

*“We're all learning, not just residents, but even us*…*I think valuing, their input…provides that kind of environment.”* (Interview 2)

*“Over time, you become closer to become friends and eventually future colleagues, because this is someone you're spending, half a day a week or a day a week for many months*…*I'm a young staff so they're not that different in age for me.”* (Interview 3)

From the onset, the more the resident shows interest, the more the supervisor invests in them, in terms of time, effort and entrustment with autonomy. Supervisors are inherently more invested in their longitudinal trainees due to the prolonged nature of the supervision, the interpersonal relationship that develops and the resident’s interest.

*“The more they seem interested, the more responsibility I give them…” (Interview 4)*

*“I'm a bit more invested in my longitudinal trainee…When someone is really wanting to learn from you, you're more excited to teach them…You're going to get to build on their skills, which is very rewarding. I would say I put in more effort at teaching in my longitudinal clinic…”* (Interview 7)

Over time, supervisors develop interpersonal relationships with their longitudinal clinic residents. This partnership strengthens throughout the clinic. This increases trust between them and enables supervisors to provide residents more autonomy. This also facilitates mentorship that extends beyond clinical training and addresses personal issues like work-life balance.

*“I'm interested in them as a person…and that…grows with time…If you're my long-term resident…We will be colleagues…I guide them…how to achieve all their goals, not just work, but also family life… That's why the longitudinal clinic can be a very rewarding experience, because you kind of grow with the person.”* (Interview 9)

Theme 2: Make clinic reflect real life by increasing volume and administrative tasks

Many participants identify a steep learning curve from the end of residency to beginning practice. A frequently identified gap is the patient volume seen by residents compared to what they would see as an attending. To address this, supervisors try to create learning opportunities that mimic practice. For example, organizing junior attending clinics, in which the resident runs clinic independently with the supervisor available as needed. This is intended to build confidence and readiness for practice.

*“A resident can be okay seeing three cases in the afternoon…then they graduate and have fifteen patients…I think the best way to encounter that…it's doing transition to a staff practice where you can act like a junior staff…Put eight patients in the afternoon nearby yourself and see how they do. You're always there as a backup support.”* (Interview 6)

Many participants discuss incorporating other practical aspects of staff work into their longitudinal clinic teaching, particularly towards the end of training. These include billing, following labs, applying for positions, as aspects that they felt were missing from their training but are important for transition to practice.

*“Another aspect…which I hadn’t seen in my training…I tell them the practical stuff…about billing*… *Towards the end of training it's more talking about the logistics of setting up your practice. How to apply for a position, where to look, who to talk to?”* (Interview 8)

Many participants identify learning value in having residents follow investigations and complete paperwork for patients they have seen to replicate practice. However, this is often not done due to time restrictions and lack of administrative support.

*“They should follow the labs and more of the admin stuff because I feel like there's learning value…that we don't learn enough…We don't realize how much the first year, a lot of the clinic stuff is 50 % paperwork…We don't show the residents that and I think that there's value in showing that*…*”* (Interview 1)

When electronic medical record (EMR) systems allow for residents to be assigned tasks, this facilitates allowing supervisors to give residents more opportunities for ownership that reflect practice.

*“It's easier when we're in the outside clinic with a good EMR, right? Because then I can…forward it to the residents so they have access to the EMR…if there's something abnormal that needs an intervention, let's say an adjustment in a dosage, I forward it.”* (Interview 11)

Theme 3: Tailor clinic to resident needs and career goals

Supervisors describe identifying residents’ needs and career-specific interests and tailoring the clinic experience accordingly over time. This involves supervisor-initiated discussions early on about what the resident hopes to gain from the experience. These include discussion of perceived knowledge gaps, lacking clinical exposures and their future practice goals.

*“At the beginning, if you establish that I want to gain more knowledge into this area or I want to be better in exams in this or I really have a deficiency in, for example, adrenal stuff, let's focus on that. It actually changes the patient populations you actually distribute to the learner.”* (Interview 6)

Once established, supervisors will tailor the clinic experiences to meet these needs. This is done by selecting cases in advance specifically for the resident to see. Some supervisors describe having periodic discussions over time to re-evaluate these goals and whether they have changed and are being met.

*“If the resident goal is eventually to go into community…the way we set up her clinic will be very differently than someone who's focusing on doing something very specific. We tend to discuss that since the beginning and throughout because I think their goals change…Some people start off thinking community, in the midway they're like, oh, I decided I want to do academia and very specifically this type*…*”* (Interview 11)

However, others acknowledge that this discussion is not re-visited over time but perhaps should be part of their supervision practice.

*“I think the idea of re-evaluating the goals of the residents during the year is something that we need to do. I usually do it at the beginning and I don't think we formally sit down and say are you getting what you want out of the clinic…I feel like I've just gotten so busy that I don't have the time to do everything the way I would want it to be but I think I have to try to start making time.”* (Interview 5)

Theme 4: Let residents make decisions and learn from them

Many participants cite that longitudinal clinic is unique in that supervisors give residents the autonomy to execute their own clinical management plans. This occurs even when that plan differs from that of the supervisor provided that patient safety is maintained. This is done to stimulate confidence and ownership.

*“Unless there's something grossly not okay with a plan I usually go with their plan*…*I think that gives them the self-confidence and the autonomy to feel okay like most times when I say something, I'm not being corrected and I think it's fair because most of the residents at that at that level are quite safe.”* (Interview 12)

Participants describe this approach as allowing residents to observe the consequences of their management decisions and as an opportunity to learn from trial and error.

*“I do try to…allow the resident to do more of…the communication, the management plan, when they're going to be following them in a few months after something was decided, as opposed to when they're not see them again. So that they can learn from their management decisions.”* (Interview 2)

*“I think the patient should increase the insulin by four units and the resident thinks two*…*I think two units may not be enough, but the proof is in the pudding. Come back in a month and we'll see whether it was enough…If you're never allowed to make a mistake, your learning is not going to be optimized. And again, a mistake, please make it clear, it's not…harmful.”* (Interview 9)

Theme 5: Let residents take patient ownership

Most participants describe residents at this stage to be afforded a baseline degree of autonomy from the onset of the longitudinal clinic not only for clinical decisions but also regarding patient ownership and clinic structure. This is based on trust earned through previous experiences with the residents and their demonstrating knowledge, confidence, judgement and initiative.

*“Most of the residents are quite independent already. So I do give them a lot of independence…several aspects, not just the patient decision…It's also a management of the clinic, how many patients they*…*I give them sort of a guidance, what's the usual amount, but then everybody can…adjust it based on that.”* (Interview 11)

Over time, the autonomy granted by supervisors progresses as the resident gains experience and continues to demonstrate knowledge and comfort. This includes having residents independently counsel patients and taking greater patient ownership. This is especially true when residents have had the opportunity to care for the same patient during multiple visits over time.

*“When you’re more established with the resident and they feel comfortable…At the beginning…I'm the one doing most of the talking…whereas later…they're having the conversation. And that's where I think it should go because it's their patient*…*”* (Interview 2)

*“The same trainee can see the same patient a few times over the 18 months…So there's a trust relationship that happens also with the patient, myself, the trainee. And it's reflective of practice of…a longitudinal specialty. You follow your patients for many years because they have chronic diseases.”* (Interview 3)

However, in cases where a resident is deemed less trustworthy, supervisors report being more hands-on and taking autonomy away by validating the resident’s assessments, verifying prescriptions and doing patient counselling themselves.

*“I just tend to be more hands-on…I'm more likely to double-check everything…I’m more likely to open the labs…the imaging versus just hearing what they said…I'll ask the patient more questions when I go back in…to confirm the information that they've given.”* (Interview 7)

While this is described as rare with subspecialty residents, when this does occur it limits the resident growth in a vicious circle. By having less responsibility and more supervision, there is less opportunity for growth. To compensate, easier, lighter caseloads are given to develop time management and self-confidence.

*“It's a self-fulfilling prophecy…because if I don't give them the autonomy, they…may not grow as much as they would have…I will contradict…the resident that I don't trust so much…Maybe it creates less of a confidence*…*”* (Interview 9)

*“In addition to…reducing the cases, maybe I tended to also to give less complex*…*From a training perspective, you see a case and then your staff and your mentor just point out all the deficiencies. I think that's very difficult to come out of that case feeling confident*…*I think it's important to rebuild the confidence when it's lacking…”* (Interview 11)

## Discussion

Our study investigated supervisor perspectives on their supervision and its changes over time in the endocrinology longitudinal clinic, using cognitive apprenticeship theory as a theoretical framework. Overall, supervisors try to encourage residents to self-regulate their learning, but also offer support and autonomy to enhance their longitudinal development. This is done by supervisors treating these residents as equal colleagues. Supervisors also try to make clinic reflect real life whenever possible by removing support and adapting pace and content to reflect practice, particularly towards the end of the training for more adept residents. Articulation and exploration are heavily relied on supervision strategies in this setting. This involves encouraging residents to articulate their needs and career goals and supervisors using these to tailor clinic experiences. Exploration involves allowing residents to execute their own management plans even if discordant with that of supervisors, providing patient safety is maintained and they trust the resident. These learner-centered supervision strategies were likely the most prominent in this setting due to the residents being more senior, similar to what has been previously described in cognitive apprenticeship theory-informed supervision of more experienced medical students [15].

Many of the supervision strategies here resemble those described in longitudinal supervision of medical students. These include encouraging the longitudinal follow up of patients, careful selection of patients and longitudinal relationships between learners and supervisors [17], [18], [19]. While these overarching ideas are similar, their application differs for residents later in training. For example, encouraging longitudinal patient follow up goes beyond seeing the progression of their clinical course. Here, it is uniquely an opportunity for residents to execute their own plans and learn from their mistakes in a supported setting. Using mistakes to enhance learning has been previously described as a useful supervision strategy [20]. This also aligns with literature reporting that residents finishing residency desire more active, self-directed learning [6], [7]. The longitudinal relationship between learner and supervisor is important to supervision in this setting as residents are described as treated as equal colleagues [17]. Over time, this involves learning from each other, growing together and discussing shared concerns of work-life balance and financial aspects of practice [21]. Given the paucity of literature on supervision of residents nearing practice, this is a unique finding with potential broad applicability to other residents near the end of training. Careful selection of patients is another common supervision strategy [17], [18], [19]. With medical students, this refers to finding patients that can form long-term connections with students however in our study, supervisors report selecting patients to meet the learning needs and career-specific interests of residents.

The study’s single-center nature could limit transferability. We attempt to mitigate this by including rich context descriptions, a strong theoretical framework and engagement in data presentation to enable broader transferability to endocrinology residents and residents in later stages of training overall [22]. Other limitations include the self-reported nature of the findings and the lack of resident perspectives. Future directions of the work include expanding to include perspectives from supervisors in other specialties and centers. Additional future directions include exploring resident perspectives of their supervision needs in longitudinal clinic as there is limited research on this topic.

## Conclusion

This study identifies supervision strategies used in the endocrinology longitudinal clinic providing suggestions on how to approach supervision of endocrinology residents over time to enhance their development. Encouraging self-directed learning is crucial to supervision in this setting. The culture of equal partnership between residents and supervisors is also important to supervision in this setting. Together, these strategies enable supervisors to support resident development into independent endocrinologists. This has the potential to further inform faculty development related to longitudinal clinical supervision.

**Funding**

The McGill University Health Centre Department of Medicine scholarship supporting research activity in medical education. The funding source had no role in study design, data collection, analysis or interpretation of data.

The McGill University Faculty of Medicine and Health Sciences Research Ethics Institutional Review Board granted ethics approval on August 7th, 2023. All subjects provided written informed consent.

## CRediT authorship contribution statement

**Isabella Albanese:** Writing – review & editing, Writing – original draft, Software, Resources, Project administration, Methodology, Investigation, Funding acquisition, Formal analysis, Data curation, Conceptualization. **Katherine Drummond:** Formal analysis. **Vanessa Tardio:** Supervision, Conceptualization. **Diana Dolmans:** Writing – review & editing, Validation, Supervision, Formal analysis, Conceptualization.

## Declaration of competing interest

The authors declare that they have no known competing financial interests or personal relationships that could have appeared to influence the work reported in this paper.

## Appendix 1 Interview guide

Opening question:

1. Can you tell me how long you’ve been working as a clinical supervisor in the endocrinology department?

Introductory questions:

2. During this time, how many residents have you supervised in the longitudinal clinic setting? Over how long did you supervise each of these residents?

Transition question:

3. The idea behind this study is to better understand clinical supervision in the endocrinology longitudinal clinic setting. There is a lot of existing literature related to clinical supervision overall. Our aim is to better understand specific factors that go into supervising endocrinology residents over a longer period of time. Can you tell me about your experience as a longitudinal clinic supervisor?

Key questions:

4. What are ways in which you set an example as a role model for residents in the longitudinal clinic? Does this change over time? If so, how? Can you give an example of this? Can you explain why?

5. What are your expectations for learners in the longitudinal clinic? How do you set these expectations?

6. How do you support residents in the longitudinal clinic? Does this change over time? If so, how? Can you give an example of this? Can you explain why?

7. Can you comment on the balance between providing this support and learner autonomy?

8. What are ways in which you observe residents in the longitudinal clinic? How does this change over time?

9. How do your observations of learners affect your teaching activities? How does this change over time?

10. How do you allow residents to demonstrate their understanding in the longitudinal clinic? How does this change over time?

11. Can you give examples of what has allowed you to trust residents in the past to give them more autonomy and less support?

12. What do you do in the scenario where you don’t trust a resident? Does this change your approach to supervision? How does this change over time?

13. What are ways in which you encourage residents to set their own learning goals in the longitudinal clinic? Is this discussed with residents directly? How does this change over time?

14. How do you encourage residents to reflect on their performance in the longitudinal clinic? How does this change over the course of the clinic?

15. Has your supervision approach been different for different endocrinology results? Can you comment on how and why?

Ending questions:

16 How does longitudinal clinic supervision differ from other clinic supervision settings?

17. Previous interviews have identified that despite activities like longitudinal clinic, there is still a big learning curve from residency to practice. Do you agree? How do you think we can minimize that gap?

18. Would you like to add anything to what we have discussed?

Additional probing questions to be used throughout interview as deemed appropriate:

• Can you elaborate on that?
• Can you provide examples of this?
• Can you explain why you think this is the case?
